# Associations between the national ‘Swap to Stop’ programme offering free vapes for smoking cessation and quit attempts in England: Results from a population‐based survey

**DOI:** 10.1111/add.70332

**Published:** 2026-03-05

**Authors:** Vera Helen Buss, Emma Beard, Lion Shahab, Erikas Simonavičius, Linda Bauld, Jamie Brown, Leonie Brose

**Affiliations:** ^1^ Department of Behavioural Science and Health University College London London UK; ^2^ Behavioural Research UK Edinburgh UK; ^3^ Department of Epidemiology and Public Health University College London London UK; ^4^ Department of Addictions Institute of Psychiatry, Psychology and Neuroscience, King's College London London UK; ^5^ National Institute of Health and Care Research (NIHR) Policy Research Unit in Addictions London UK; ^6^ Usher Institute University of Edinburgh Edinburgh UK

**Keywords:** England, health inequities, health policy, interrupted time series analysis, tobacco smoking, vaping

## Abstract

**Background and aims:**

Vapes are effective for smoking cessation. The UK Government launched the Swap to Stop initiative in England in December 2023, aiming to encourage people to quit smoking by providing free vape starter kits alongside behavioural support. This study aimed to assess the association between the introduction of Swap to Stop and the proportion of people in England who tried to quit smoking using vapes in the past year.

**Design:**

Data came from the Smoking Toolkit Study, a monthly cross‐sectional population‐based survey. The primary analysis used an interrupted time‐series approach based on Autoregressive Integrated Moving Average (ARIMA) regression models.

**Setting:**

Telephone interviews with people residing in private households in England between December 2021 and December 2024.

**Participants:**

People aged ≥16 years who smoked in the past year.

**Measurements:**

The outcome was vape use during past‐year quit attempts. The intervention effect was included as a step change in December 2023 to indicate the start of the Swap to Stop programme. The model also included a dummy variable to adjust for above‐inflation tobacco tax increases.

**Findings:**

The primary analysis indicated that the introduction of Swap to Stop in December 2023 was associated with a 1.5 absolute percentage point increase (adjusted B = 0.015, 95% confidence interval = 0.005–0.025) in the proportion of people in England using vapes in past‐year quit attempts that persisted to December 2024.

**Conclusions:**

The introduction of Swap to Stop (which provides free vape starter kits with behavioural support to quit smoking) in England appears to be associated with a statistically significant increase in quit attempts using vapes.

## INTRODUCTION

Vapes have been shown to be an effective smoking cessation tool, increasing the number of people who quit by approximately 4 per 100 compared to nicotine replacement therapy [1]. In England, where vaping for smoking cessation is recognised as one component of tobacco control efforts, approximately 1 in 3 adults report using vapes during a quit attempt, based on data collected between 2013 and 2023 [2]. In 2023, the United Kingdom (UK) Government announced a new ‘Swap to Stop’ initiative to help people quit smoking as one part of a series of measures aimed at decreasing smoking prevalence to 5% or less by 2030 [3].

The Swap to Stop initiative provides free vape starter kits alongside behavioural support [4]. The government is funding the provision of kits to almost 1 in 5 people who smoke, particularly those from populations with higher smoking rates, across England, initially until March 2025 with an extension for another year announced in March 2025 [5]. The programme has collaborated with job centres, homeless centres, addiction treatment centres, outreach mental health services/teams, social housing providers, local Stop Smoking Services and other services within the National Health Service (e.g. maternity services) that are providing vape starter kits as part of their existing cessation services [4]. By October 2024, 218 expressions of interest (EoIs) had been submitted by service providers or local authority‐led partnerships, representing at least 125 of England's 317 local authorities [6]. Some submissions covered multiple local authorities, while others involved multiple EoIs from the same authority for different types of service providers [6]. Providers could choose vape starter kits from a procurement platform for up to £40 per unit and offer vouchers redeemable at participating online or physical vape shops [7].

The combination of vapes and behavioural support can help address the physical and psychological aspects of nicotine addiction. Vapes deliver nicotine effectively while exposing people to fewer harmful toxicants than cigarettes and are, therefore, a useful tool to transitioning away from smoking. Behavioural support provides the necessary guidance to start using vapes and reinforcement to stay motivated and manage cravings [8].

A programme of giving out free vapes was piloted in 2018 in a socially deprived area of England via Stop Smoking Services in the community or in pharmacies. At 4‐weeks follow‐up, 60% of participants still engaged with the programme and, overall, 37% were confirmed to have quit cigarette smoking (57% of those who were followed up in the community and 76% of those who were followed up in the pharmacy) [9].

This study is part of a wider evaluation [7] of the Swap to Stop programme and aimed to assess its initial impact on rates of quit attempts using vapes among adults who smoke. The current study used two different approaches: an interrupted time series approach using aggregated data from England and a difference‐in‐differences approach using individual‐level data comparing changes England with those in Scotland and Wales where Swap to Stop was not implemented.

The research questions (RQ) were:

1. Has there been a detectable change in quit attempts using vapes among adults who smoked in the past year in England since December 2023 when the Swap to Stop programme started?
2. How do changes in quit attempts using vapes among adults who smoked in the past year in England before and after the start of Swap to Stop compare to changes in Scotland and Wales over the same period?

## METHODS

### Study design and participants

This study used data from a population‐based, repeat cross‐sectional survey, the Smoking Toolkit Study [10]. Data were collected monthly via telephone interviews between December 2021 and December 2024 by Ipsos Mori. Each month, adults 16 years or older were surveyed, approximately 1700 in England, 450 in Scotland and 300 in Wales (in December 2021, 16‐ and 17‐year‐olds were not included because of temporary coronavirus disease‐related changes in data collection). Households were selected using a hybrid of random location and quota sampling. Interviews were conducted with each one household member until quotas, determined from factors influencing the probability of being at home (e.g. age, gender, working status), were fulfilled [10]. The research team had only access to de‐identified data. The manuscript followed the Strengthening the Reporting of Observational Studies in Epidemiology (STROBE) statement [11]. The study protocol was pre‐registered on the Open Science Framework (https://osf.io/d8jxp/).

For RQ1, we included participants who smoked in the past year living in England. For RQ2, we included participants who smoked in the past year living in Great Britain (i.e. England, Scotland and Wales) and divided them into the ‘intervention’ group (i.e. those from England) and the ‘control’ group (i.e. those from Scotland and Wales).

We identified those who smoked in the past year based on the question ‘Which of the following best applies to you?’ The answer options included (i) ‘I smoke cigarettes (including hand‐rolled) every day’; (ii) ‘I smoke cigarettes (including hand‐rolled), but not every day’; (iii) ‘I do not smoke cigarettes at all, but I do smoke tobacco of some kind (eg. Pipe, cigar or shisha)’; (iv) ‘I have stopped smoking completely in the last year’; (v) ‘I stopped smoking completely more than a year ago’; (vi) ‘I have never been a smoker (i.e. smoked for a year or more)’; and (vii) ‘Do not know’. Those who replied answer options (i), (ii) or (iii) were classified as having smoked in the past year.

### Sample size

For RQ1, which is based on autoregressive integrated moving average (ARIMA) regression analysis, we conducted a simulation‐based power calculation for an interrupted time series analysis [12] using an ARIMA(1,0,0) model with a step change and autoregressive (AR(1)) coefficient = 0.5. SD of the white noise was set to 2. We simulated 1000 datasets, each spanning 36 months, with an intervention introduced at month 24. The baseline prevalence was set at 12.5%, with a step increase to 14.4% following the intervention (pre‐registered calculation used the wrong baseline prevalence). Based on these parameters, our analysis demonstrated that the study design achieved 88% power at a significance level of 0.05.

### Outcome variables

We calculated the monthly prevalence of quit attempts using vapes among people who smoked in the past year by dividing the number of people who reported using vapes in the most recent quit attempt by the number of all past‐year smokers each month. We used the following question to assess whether people tried to quit: ‘How many serious attempts to stop smoking have you made in the last 12 months? By serious attempt I mean you decided that you would try to make sure you never smoked again. Please include any attempt that you are currently making and please include any successful attempt made within the last year.’ In a follow‐up question, those who reported at least one quit attempt were asked: ‘Which, if anything, of the following did you try to help you stop smoking during the most recent serious quit attempt?’ Those who answered ‘electronic cigarette or vaping device’ or ‘Juul’ were classified as having made a past‐year quit attempts using vapes.

The main analyses refer to quit attempts within the past 12 months, which could mean that these occurred before the start of Swap to Stop and are not directly related to it. Therefore, we included a sensitivity analysis in which we restricted the outcome to using vapes in past‐month quit attempts. This could minimise the problem of misattributing quit attempts to Swap to Stop, but the study may not have sufficient power to measure an effect on quit attempts within the past‐month. Vape use in past‐month quit attempts was determined based on the following question among those reporting a past year quit attempt: ‘How long ago did your most recent serious quit attempt start? By most recent, we mean the last time you tried to quit.’, with the answer options: (i) ‘In the last week’; (ii) ‘More than a week and up to a month’; (iii) ‘More than 1 month and up to 2 months’; (iv) ‘More than 2 months and up to 3 months’; (v) ‘More than 3 months and up to 6 months’; (v) ‘More than 6 months and up to a year’; and (vi) ‘Do not know’. All those who replied (i) or (ii) were classified as having tried to quit in the past month. We, then, calculated the monthly prevalence of past‐month quit attempts using vapes among people who smoked in the past year by dividing the number of people who reported using vapes in a past‐month quit attempt by the number of all past‐year smokers each month.

### Covariates

Time was measured in months, representing the monthly survey wave and was coded from 1 (December 2021) to 49 (December 2024). Furthermore, we included a dummy variable to measure the step change that was coded as 0 from December 2021 until November 2023 (pre‐intervention period) and 1 from December 2023 until December 2024 (intervention period). We chose a step change to model the intervention as we anticipated that the introduction of the programme resulted in an immediate, sustained increase in the number of people making a quit attempt using vapes. We selected December 2023 as the point when the Swap to Stop programme started, anticipating any change around this time was likely because of the announcement and media coverage of the programme rather than vape kits necessarily all being immediately distributed. However, some services may have already started giving out vapes to service users as early as December 2023, for example, by using existing supplies, before distributing the exact devices funded by the government under Swap to Stop.

For RQ2, we used a further dummy variable to categorise participants into the ‘intervention’ and ‘control’ group. Those living in England who were ‘exposed’ to the Swap to Stop programme were coded as 1 and those living in Scotland and Wales as 0. Furthermore, we included a dummy variable to the model to adjust for seasonality that was coded from 1 (i.e. January) to 12 (i.e. December) and modelled using a smoothing term with a cyclic cubic spline function [13]. Covariates included age, gender and socio‐economic status. Age was included as a numerical variable modelled using restricted cubic splines [14]. Gender was included as a binary variable (women or men). For descriptive statistics, we also included people who identified as non‐binary, but because of small numbers these were not included in the analysis for RQ2. Socio‐economic status was included as a binary variable, grouping people into more and less advantaged based on occupational social grade (ABC1—more advantaged, which includes high, intermediate, supervisory, clerical and junior managerial, administrative or professional; C2DE—less advantaged, which includes skilled, semiskilled and unskilled manual workers, state pensioners, casual or lowest grade workers and unemployed with state benefits only) [15].

Furthermore, we adjusted both analyses for tobacco‐related tax increases that occurred during the study period. We created a dummy variable coded as 1 for months in which an above‐inflation tax increase was implemented (each November, plus in April 2023), and 0 otherwise [16, 17].

### Analysis

The analysis was conducted in RStudio (version 2022.07.2, R version 4.2.1). The level of significance was set to 0.05 and 95% CI are provided. Number of missing values for each collected variable required for the analysis is reported in Table S1. We conducted a complete case analysis. A small number of participants (2.0%, *n* = 285) provided their age only as a range rather than an exact value. For these, we imputed their age using the median for their respective age range. This approach was not specified in the protocol, and we tested the robustness of the approach by also reporting participant characteristics when excluding participants without exact age value (Table S2). The age variable was only used to describe the sample characteristics and as a covariate for RQ2. It did not have an impact on the results of RQ1.

For RQ1, data were aggregated monthly and weighted using raking [18] to match the population of England based on socio‐demographic characteristics. For the weighting, we used the R package ‘*survey*’ for complex survey analysis, which is designed to maintain integrity of the sample design when analysing subgroups [18, 19]. The weighted sample size for each survey wave is presented in Table S3.

Data were analysed using ARIMA regression models using the *'* *TSA* *S* *'* package in R [20], following recommended procedures for time series analysis [21, 22]. In short, first, we tested whether outliers were presented in the data set using boxplots and the ‘*tsoutlier*’ function from the ‘*forecast*’ package [23], but no outliers were identified. Second, we plotted the autocorrelation function (ACF) and the partial ACF and found no indication of autocorrelation present in the data. Third, we checked whether non‐seasonal or seasonal differencing was required to make the time series stationary by visually inspecting it and using unit root tests. The results suggested that the time series was stationary without requiring differencing. Fourth, we selected the number of AR (i.e. autoregression) and MA (i.e. moving average) terms based on the model fit using primarily the Akaike information criterion and as a secondary indicator for robustness the Bayes information criterion. In both cases, the smallest value indicates the best fitting model. Fifth, using the model with the smallest Akaike information criterion, we plotted the residuals time series, residuals ACF and the residuals histogram and used the Ljung‐Box test to check for additional autocorrelation. We selected the best fitting model without adjustment and finally added the dummy variable for above‐inflation tax increases to it. Unadjusted and adjusted models are reported.

For RQ2, data were weighted to match the population of Great Britain. We used a generalised additive model (GAM) to conduct a difference‐in‐differences analysis. We used the *‘gam’* function of the ‘*mgcv*’ package with a quasi‐binomial family using the log as a link function [24]. We reported unadjusted and adjusted (age, gender, socio‐economic position and tobacco‐related tax increases) results. One of the requirements for the difference‐in‐difference analysis is the parallel‐trends assumption (i.e. requiring similar rates of using vapes in quit attempts in England before Swap to Stop as in Scotland and Wales). To assess whether the assumption was met, we plotted the modelled trends derived from the GAM. Since the assumption was violated because the pre‐trend in England differed from the trend in Scotland and Wales, the difference‐in‐differences approach proved to be unsuitable. Therefore, the results for RQ2 are only presented in the supplementary file. We explored different statistical approaches, including propensity score matching and synthetic controls, to make the sample of Scotland and Wales an appropriate control group for the analysis. However, none of the approaches were suitable. For propensity score matching, substantial differences in covariates and the much smaller size of the control group made finding matches difficult. Moreover, the algorithm tended to pull England's matched sample toward the control characteristics, which distorted its pre‐intervention trend. Synthetic control methods require multiple donor units, but we only had two (Scotland and Wales), both with considerable smaller samples than England, leaving insufficient data to replicate England's pre‐interventions trajectory.

Therefore, the Results section focusses on the primary analysis using ARIMA modelling.

We conducted several sensitivity analyses. First, we tested a step change starting in April 2024 rather than December 2023, to assess whether the intervention effect may have started with a delay after the start of the programme roll‐out. Second, as stated above, we ran the analysis with the outcome being prevalence of vape use in past‐month quit attempts. More details of the methods and the model selection processes are provided in the Supporting information and the pre‐registered protocol (https://osf.io/uaxc8/).

## RESULTS

Over the entire period, the prevalence of using vapes in past‐year quit attempts was 13.9% (95% CI = 13.1–14.6) and in past‐month quit attempts 10.0% (95% CI = 9.4–10.7) among people who smoked in the past year in England. Further participant characteristics are presented in Table 1.

**TABLE 1:** Weighted characteristics of participants with complete data who smoked in the past year (N_unweighted_ = 13 619).

	England	Scotland and Wales
Sample size, *n*	13 678	1775
Age, median (IQR)	38 (28–53)	48 (33–62)
Women, % (95% CI)	45.6 (44.6–46.7)	45.7 (43.9–47.5)
Men, % (95% CI)	53.1 (52.1–54.2)	51.8 (50.0–53.7)
Non‐binary, % (95% CI)	1.2 (1.0–1.4)	2.5 (1.8–3.2)
Less advantaged socio‐economic position, % (95% CI)	57.3 (56.3–58.3)	60.2 (58.5–61.9)
Past‐year quit attempt, % (95% CI)	36.5 (35.5–37.6)	32.7 (31.0–34.4)
Past‐year quit attempt using vapes, % (95% CI)	13.9 (13.1–14.6)	11.5 (10.3–12.7)
Past‐month quit attempt, % (95% CI)	26.0 (25.0–26.9)	23.7 (22.2–25.3)
Past‐month quit attempt using vapes, % (95% CI)	10.0 (9.4–10.7)	8.3 (7.3–9.4)

Abbreviation: IQR, interquartile range.

### Change in quit attempts using vapes in England

**FIGURE 1:**
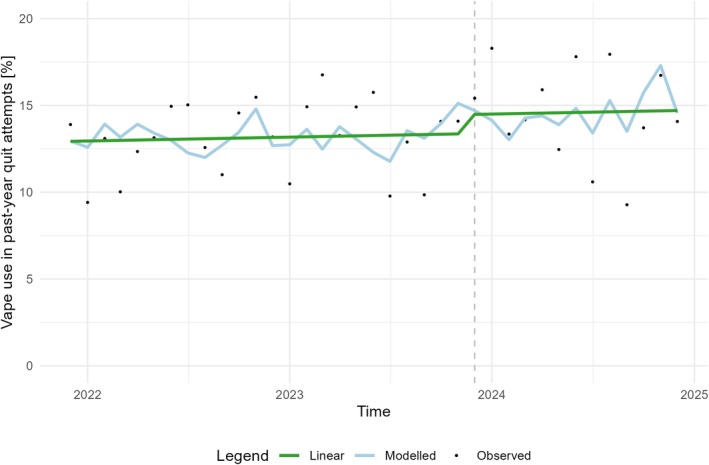
Monthly estimates for proportion of people who smoked in the past year in England who used vapes in past‐year quit attempts. The black dots indicate the observed estimates, the blue line indicates the modelled estimates from the ARIMA model, the green line indicates the linear trend with a step change in December 2023, and the vertical grey dashed line indicates the time point when the Swap to Stop programme was implemented.

Figure 1 shows the observed estimates for prevalence of vape use in past‐month quit attempts among people who smoked in the past year in England across time as well as the modelled estimates from the adjusted ARIMA model and a modelled linear trend with a step change in December 2023. The β coefficient for the step change in December 2023 from the ARIMA model was statistically significant (Table 2; for more details on the final model and selection process, see Figures S1–S3 and Tables S4–S7). When adjusting for above‐inflation tobacco tax increases that occurred during the study period, the model indicated that the introduction of the Swap to Stop programme resulted in a sustained 1.5 percentage point increase in the average proportion of past‐year quit attempts using vapes made by people who smoked in the past year in England.

**TABLE 2:** Results for best fitting models for the association of the Swap to Stop programme and prevalence of quit attempts using vapes among adults in England who smoked in the past year.

Outcome	Modelled^a^ intervention effect	Unadjusted		Adjusted^b^	
		B (95% CI)	*P*‐value	B (95% CI)	*P*‐value
Past‐year quit attempt using vapes	Step change in December 2023	0.014 (0.003–0.026)	0.017	0.015 (0.005–0.025)	0.004
Past‐year quit attempt using vapes	Step change in April 2024	0.008 (−0.007 to 0.023)	0.299	0.008 (−0.006 to 0.022)	0.263
Past‐month quit‐attempt using vapes	Step change in December 2023	0.009 (−0.003 to 0.021)	0.151	0.009 (−0.002 to 0.020)	0.096

Abbreviation: ARIMA, autoregressive integrated moving average.

^a^ Using an ARIMA(0,0,1) model.

^b^ Adjusted for tobacco‐related tax increases. For full model specifications, see Tables S5–S7.

Assuming that, of the roughly 47 million people aged 16 years or older living in England in 2024 [25], 17.8% smoked in the past year, this corresponds to approximately 8 366 000 individuals. If an additional 1.5% of these individuals attempted to quit in the past year using vapes as a result of Swap to Stop, this would correspond to an estimated 125 490 additional people making a quit attempt with a vape since its introduction, with the true number likely falling between 41 830 and 209 150 (based on 95% CI of the step change: 0.5–2.5 percentage points).

Both sensitivity analyses—using past‐month quit attempts as the outcome and using a step change in April 2024 to model the intervention effect—produced estimates that were consistent with the primary analysis, but were not significant. For both outcomes, the measured step change effect was smaller than for the primary analysis, which would be expected if the policy already started taking effect at the beginning in December 2023 rather than with a delay in April 2024. Furthermore, the intervention effect on past‐month quit attempts is logically expected to be smaller than on past‐year quit attempts, but the uncertainty may be explained by the small sample size.

### Changes in England compared with Scotland and Wales

The prevalence of using vapes in past‐year quit attempts was 11.5% (95% CI = 10.3–12.7) and 8.3% (95% CI = 7.3–9.4) in Scotland and Wales, lower than in England. Generally, a higher proportion of those residing in England reported making a quit attempt in the past year (36.5%, 95% CI = 35.5–37.6) than in Scotland and Wales (32.7%, 95% CI = 31.0–34.4). The median age in Scotland and Wales was noticeably higher than in England, and a higher proportion of those living in Scotland and Wales identified as non‐binary and was from a less advantaged socio‐economic position (Table 1).

These differences, alongside a significant positive association between age and vape use in quit attempts (see Figures S4‐S5), meant Scotland and Wales were not a suitable control group for the difference‐in‐differences approach, but we present the pre‐specified analyses in the Supporting information (Table S8 and Figure S6).

## DISCUSSION

The primary analysis indicated that the introduction of the Swap to Stop programme in December 2023 was associated with a sustained 1.5 percentage point increase in people in England using vapes in attempts to quit smoking in the past year. This increase equates to approximately 125 000 additional quit attempts using vapes, which may be attributable to Swap to Stop (in comparison, overall, there were ~3 million past‐year quit attempts in 2024). The two sensitivity analyses were consistent with the primary result, but potentially uncertain because of lack of statistical power (i.e. past‐month quit attempts) or because the effect was detectable already at the start of Swap to Stop rather than with a few months delay (i.e. step change in April 2024). Although the step change was immediate, evidence suggests that many providers had not yet received vape kits from the government in December 2023 [6], implying that the immediate effect may be sensitive to an initial media push. The secondary analysis was not suitable for detecting an effect because the pre‐intervention trends differed between England and Scotland and Wales, but it is a pre‐requisite for the difference‐in‐differences analysis that the trends run in parallel.

### Comparison with previous studies

Although we did not assess quit success rates due to insufficient power, a randomised controlled trial at three local Stop Smoking Service sites, which offered participants either vapes or nicotine replacement therapy along with behavioural support, showed that vapes were significantly more effective than nicotine replacement therapy and providing vape starter kits had the potential to increase quit success rates [26]. Similarly, a randomised controlled trial conducted in Australia showed that vapes were more effective than nicotine replacement therapy for smoking cessation among socially disadvantages populations [27]. Building on prior evidence that 1 in 5 participants quit smoking when provided with free vapes and support [9], and given the established effectiveness of vapes for smoking cessation [1], the observed increase in their use for quit attempts suggests that the Swap to Stop programme has likely had a positive impact on smoking cessation. Although the overall effect observed in this study may appear modest, even small treatment effects for smoking cessation are clinically meaningful because the health benefits of quitting smoking are enormous [28]. However, it is also noteworthy that some of these additional 125 000 quit attempts may have occurred regardless of Swap to Stop, albeit potentially unaided or with less effective methods. It is also unclear how many of those who attempted to quit might (i) have started using vapes alongside cigarettes; (ii) switched completely from smoking to vaping; or (iii) only vaped in the short‐term until they completely stopped using nicotine altogether. The effectiveness of the scheme will be further assessed in later stages of the ongoing evaluation of the Swap to Stop programme.

Beyond directly impacting quit attempts and successes via the vape starter kits given out by Stop Smoking Services, the Swap to Stop programme has the potential to improve public misperceptions about the relative health harms of vapes compared to combustible cigarettes and increase awareness of vapes as quitting aids. A study from 2014 to 2023 showed that among people who smoked in England, harm perceptions of vapes have considerably deteriorated over time, with approximately a quarter of participants thinking that vapes are more harmful than combustible cigarettes in 2023 [29]. By promoting the use of vapes as quit aids via Swap to Stop and, respectively, Stop Smoking Services, more adults who currently smoke could be encouraged to use them in their cessation attempts [7].

From a public health perspective, ideally, people who quit smoking using vapes would eventually abstain from both vaping and smoking. A systematic review of stop smoking interventions that included the provision of vapes found that, on average, 70% of participants who had quit smoking using vapes still used them after 6 months or longer [30]. Heterogeneity between included studies seemed to suggest that, in trials using newer devices, participants continued using vapes for a longer time [30]. However, sustained vapes use might help prevent smoking relapses [31].

There is also a question around who might benefit most from such interventions as the Swap to Stop initiative. A study providing brief smoking advice, together with vape starter kits and referral to Stop Smoking Services in emergency departments in Great Britain, suggested that those who were less experienced with vapes might have been particularly receptive to the intervention [31]. In a qualitative study including interviews with Stop Smoking Service providers and users, one theme that emerged was the importance of receiving behavioural support alongside the vape kit, especially for those with previous unsuccessful attempts [32]. This support allowed service providers and users to explore reasons for unsuccessful attempts and develop strategies to make future attempts more likely to succeed [32]. Similarly, in a study providing stop smoking advice and vapes in pharmacies, behavioural support was identified as a key facilitator by service users [33]. The Swap to Stop programme consists of a combination of free vape kits and behavioural support. The programme also led to facilities that previously offered no such services now offering stop smoking support. This may have increased the number of quit attempts in groups not reached by traditional Stop Smoking Services. As part of the wider evaluation of the programme, qualitative research will explore perceptions and experiences of the intervention from the perspective of service providers and users [7].

### Strengths and limitations

This study is the first to investigate the impact of a first‐of‐its‐kind government funded initiative offering free vape kits and support on rates of quit attempts in which vapes are used. A strength of the study is that it used data from a representative population‐based survey. Among the limitations are that it only used 1 year of data after the start of the programme. Therefore, the study assessed only medium‐term and not long‐term changes, but this analysis is vital to inform continued funding policy decisions. Another limitation is that the parallel trends assumption required for the difference‐in‐differences analysis was violated, which meant that this method was unsuited for the study. Because we conducted interrupted time series analysis on repeat cross‐sectional data, the observed step change should be interpreted as temporal association rather than definitive evidence of causality. Although we accounted for above‐inflation tobacco tax increases and are not aware of other relevant policies during the study period, media coverage of potential measures, such as the Tobacco and Vapes Bill debated at the time, could have influenced the results [3].

Furthermore, it is noteworthy that we only assessed use of vapes in quit attempts, but not whether the quit attempts were successful. Additionally, we included a sensitivity analysis using past‐month quit attempts as the outcome rather than past‐year quit attempts, because someone could have reported a past‐year quit attempt that occurred before the implementation of Swap to Stop and would, therefore, not be attributable to the intervention. However, when using past‐month quit attempts we were unable to detect a significant effect, likely because of insufficient power.

As Swap to Stop was designed to particularly target disadvantaged groups, including groups who do not live in private households, such as people experiencing homelessness or individuals residing in institutions, this study may underestimate the true effect of the programme on quit attempts using vapes [34]. A further limitation is that we did not include subgroup analyses to assess potential differences by socio‐economic position because of insufficient power. Therefore, future studies should prioritise evaluating equity impacts, given the programme's stated aims.

## CONCLUSIONS

This study, using an interrupted time series approach, detected significant increase in past‐year quit attempts using vapes among people who smoked in the past year in England in December 2023. This increase may be attributable to the start of the Swap to Stop campaign encouraging more people who smoke to attempt quitting using vapes. Policymakers in England should consider continued funding for the programme, and other countries could consider the option of similar models if this is possible within their national tobacco control strategies.

## AUTHOR CONTRIBUTIONS

**Vera Helen Buss**: Conceptualisation; data curation; formal analysis; methodology; visualisation; writing—original draft. **Emma Beard**: Conceptualisation; validation; methodology; writing—review and editing. **Lion Shahab**: Conceptualisation; methodology; funding acquisition; writing—and editing. **Erikas Simonavičius**: Conceptualisation; methodology; writing—review and editing. **Linda Bauld**: Conceptualisation; methodology; funding acquisition; writing—review and editing. **Jamie Brown**: Conceptualisation; data curation; methodology; validation; funding acquisition; writing—review and editing. **Leonie Brose**: Conceptualisation; methodology; funding acquisition; writing—review and editing.

## DECALARATION OF INTERESTS

J.B. has received unrestricted research funding from Pfizer and J&J, who manufacture smoking cessation medications. L.S. has received honoraria for talks, unrestricted research grants and travel expenses to attend meetings and workshops from manufactures of smoking cessation medications (Pfizer; J&J) and has acted as paid reviewer for grant awarding bodies and as a paid consultant for health care companies. All authors declare no financial links with the tobacco or vaping industry or their representatives.

## PRE‐REGISTERED HYPOTHESIS

The study protocol was pre‐registered on the Open Science Framework (https://osf.io/d8jxp/).

## ETHICS STATEMENT

The University College London Ethics Committee granted ethical approval for the Smoking and Alcohol Toolkit Study (ID 0498/001). Participants provided informed consent before taking part in the study.

## Supporting information


**Data S1.** Supporting information.

## Data Availability

The data that support the findings of this study are openly available in Open Science Framework at https://osf.io/d8jxp/, reference number DOI: 10.17605/OSF.IO/D8JXP.
