# Supplementary material for: Associations between the national ‘Swap to Stop’ programme offering free vapes for smoking cessation and quit attempts in England: Results from a population‐based survey
Source: Addiction. 2026 Mar 5;121(6):1557–65. doi: 10.1111/add.70332 (PMC13155265; doi:10.1111/add.70332)
Supplement: Supplementary file 1 — Data S1. Supporting information. [file ADD-121-1557-s001.pdf]

SUPPLEMENT

Article: Associations between the national 'Swap to Stop' programme offering free vapes for smoking cessation and quit attempts in England: results from a population-based survey

Contents

Methods 2
- Missing values and outliers 2
- Determining trends and stationarity 2
- General note regarding the interpretation of the fitted ARIMA models 2
- R Packages (including their dependencies) used for analyses 3
- Robustness check for imputing exact age values by using median of age range 3

Time-series analysis – vape use in past-year quit attempts 5
- Sensitivity analysis 1a: alternative step change 7
- Sensitivity analysis 1b: alternative outcome 7

Association between age and using vapes in quit attempts 8

Difference-in-differences – vape use in past-year quit attempts 9

References 11

Methods

Missing values and outliers

If the exact age was missing, but participants stated the age band they belonged to, we imputed their age with the median age based on those in their age band that provided the exact age. Based on boxplots and the 'tsoutliers' function from the 'forecast' package [1] we did not identify any outliers.

Table S1: Variables used for the analysis and the number (%) of missing values for each of them ($N_{unweighted}$=14,594).

Variable	Missing values n (%)
Exact age	285 (2.0)
Age banded	4 (0.0)
Gender	84 (0.1)
Social grade	0 (0)
Quit attempt in past year	703 (4.8)
Quit attempt in past month	0 (0)
Using vapes in past-year quit attempt	0 (0)
Using vapes in past-month quit attempt	0 (0)

Determining trends and stationarity

We visually inspected the plotted time series and used unit root tests to determine whether non-seasonal or seasonal differencing were required for stationarity. For the visual inspection, we plotted each time series and additionally decomposed them into three components: a trend, a seasonal, and a random component. We inspected the plots for signs of non-stationarity by looking for evidence of a trend in the mean, variance, autocorrelation, or seasonality [2, 3]. Additionally, we used the Augmented Dickey-Fuller test [4] (ndiffs function in R) to check for the number of non-seasonal differences and the seasonal unit root test according to Wang, Smith & Hyndman [5] (nsdiffs function in R) to check the number of seasonal differences required to make the time series stationary.

General note regarding the interpretation of the fitted ARIMA models

The AR term captures the relationship between an observation in the time series and a certain number of lagged observations. A positive AR coefficient (ϕ) indicates that past values of the time series have a positive linear relationship with the current value. In other words, if ϕ is positive, an increase in past values leads to an increase in the current value. Conversely, a negative AR coefficient suggests an inverse relationship, where an increase in past values leads to a decrease in the current value. The order of the AR term (p) indicates the number of lagged observations considered in the model. The MA term captures the relationship between the current value of the time series and past forecast errors (residuals). A positive MA coefficient (θ) indicates that past forecast errors have a positive linear relationship with the current value. In other words, if θ is positive, an increase in past forecast errors leads to an increase in the current value. Similarly, a negative MA coefficient suggests an inverse relationship, where an increase in past forecast errors leads to a decrease in the current value. The order of the MA term (q) indicates the number of past forecast errors considered in the model.

R Packages (including their dependencies) used for analyses

- tidyverse [6]
- survey [7]
- tibbletime [8]
- forecast [9]
- ggplot2 [10]
- tseries [11]
- TSA [12]
- zoo [13]
- splines [14]
- mgcv [15]

Robustness check for imputing exact age values by using median of age range

Table S2: Weighted characteristics of participants with complete data who smoked in the past year when not imputing exact age values based on age range ($N_{unweighted}$=13,375).

	England	Scotland & Wales
Sample size, n	13462	1743
Age, median (IQR)	38 (28, 53)	48 (32, 62)
Women, % (95% CI)	45.6 (44.5, 46.7)	45.7 (43.8, 47.5)
Men, % (95% CI)	53.2 (52.1, 54.3)	52.0 (50.1, 53.8)
Non-binary, % (95% CI)	1.2 (1.0, 1.4)	2.4 (1.6, 3.1)
Less advantaged socioeconomic position, % (95% CI)	57.4 (56.3, 58.4)	60.6 (58.8, 62.3)
Past-year quit attempt, % (95% CI)	36.6 (35.5, 37.6)	32.9 (31.1, 34.6)
Past-year quit attempt using vapes, % (95% CI)	13.9 (13.1, 14.6)	11.6 (10.4, 12.8)
Past-month quit attempt, % (95% CI)	26.0 (25.1, 27.0)	23.8 (22.2, 25.4)
Past-month quit attempt using vapes, % (95% CI)	10.0 (9.3, 10.7)	8.4 (7.3, 9.4)

As the estimates in Table S2 are very similar to the estimates in Table 1 (main manuscript), we can assume that it is acceptable to impute exact age values by using the median of the age range if provided.

Table S3: Weighted sample size of people smoking in the past year for each survey wave in England.

Wave	Sample size (95% CI)
1	294 (256, 333)
2	276 (246, 312)
3	312 (274, 350)
4	264 (225, 302)
5	295 (258, 331)
6	309 (270, 347)
7	282 (245, 319)
8	265 (228, 301)
9	296 (257, 335)
10	312 (280, 362)
11	271 (234, 309)
12	333 (294, 372)
13	293 (254, 332)
14	292 (256, 328)
15	288 (250, 326)
16	289 (251, 327)
17	299 (261, 336)
18	304 (266, 343)
19	330 (290, 370)
20	298 (262, 334)
21	319 (280, 358)
22	321 (283, 360)
23	286 (249, 323)
24	282 (245, 320)
25	272 (237, 308)
26	296 (257, 335)
27	286 (248, 324)
28	299 (258, 341)
29	280 244, 316)
30	338 (298, 378)
31	312 (273, 352)
32	293 (252, 334)
33	297 (285, 337)
34	315 (275, 355)
35	268 (232, 305)
36	273 (235, 312)
37	280 (241, 320)

Time-series analysis – vape use in past-year quit attempts

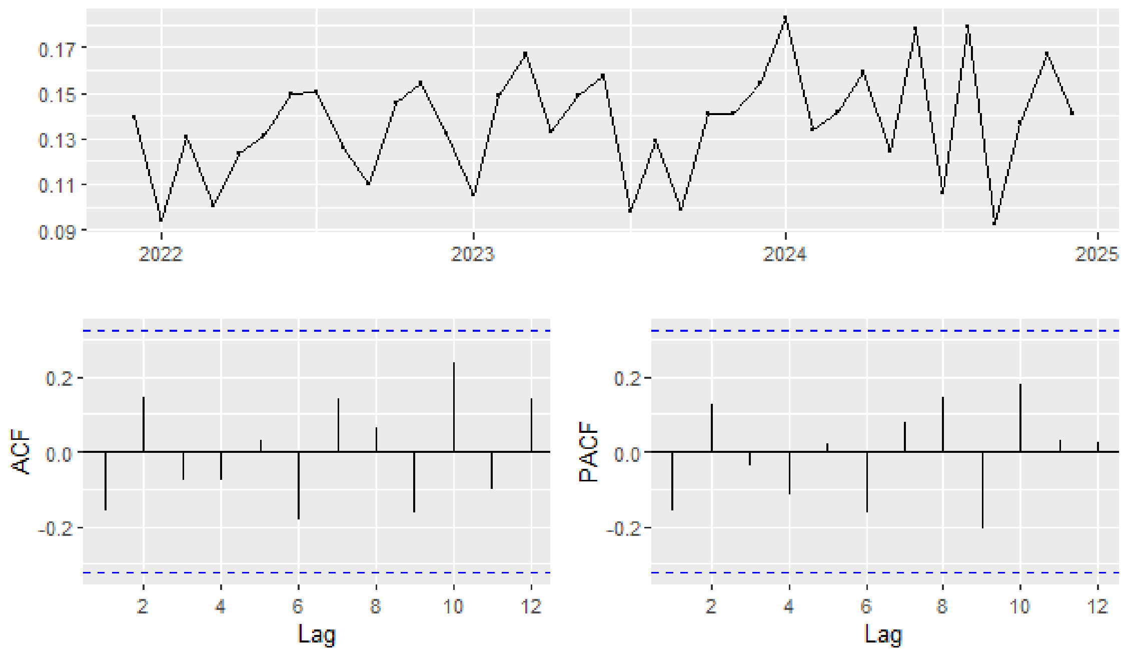

Figure S1: Time series, auto-correlation function (ACF), and partial auto-correlation function (PACF) graphs of prevalence of past-year vape use in quit attempts as the outcome variable.

- Augmented Dickey-Fuller test shows no differences required to make series stationary.
- Seasonal unit root test shows no seasonal differences required to make series seasonally stationary.

Table S4: Akaike information criterion (AIC) and Bayesian information criterion (BIC) for different ARIMA models for prevalence of past-month vape use in quit attempts as the outcome variable (unadjusted).

ARIMA	AIC	BIC
(1,0,0)	-168.9	-162.4
(0,1,0)	-131.3	-128.1
(0,0,1)	-168.6	-162.1
(1,1,0)	-149.7	-144.9
(1,0,1)	-167.0	-159.0
(0,1,1)	-159.0	-154.1
(1,1,1)	-167.0	-159.0
(2,0,0)	-159.1	-152.6
(2,1,0)	-151.7	-145.3
(2,0,1)	-165.0	-155.4
(2,1,1)	-157.4	-149.3
(0,0,2)	-166.8	-158.7
(1,0,2)	-165.1	-155.4
(0,1,2)	-158.7	-152.3
(1,1,2)	-157.3	-149.2
(2,1,2)	-155.4	-145.7

- ➔ Smallest AIC and BIC for ARIMA(1,0,0), then ARIMA(0,0,1)
- ➔ Go with ARIMA(0,0,1) as residuals from regression look more normally distributed with this model than ARIMA(1,0,0)

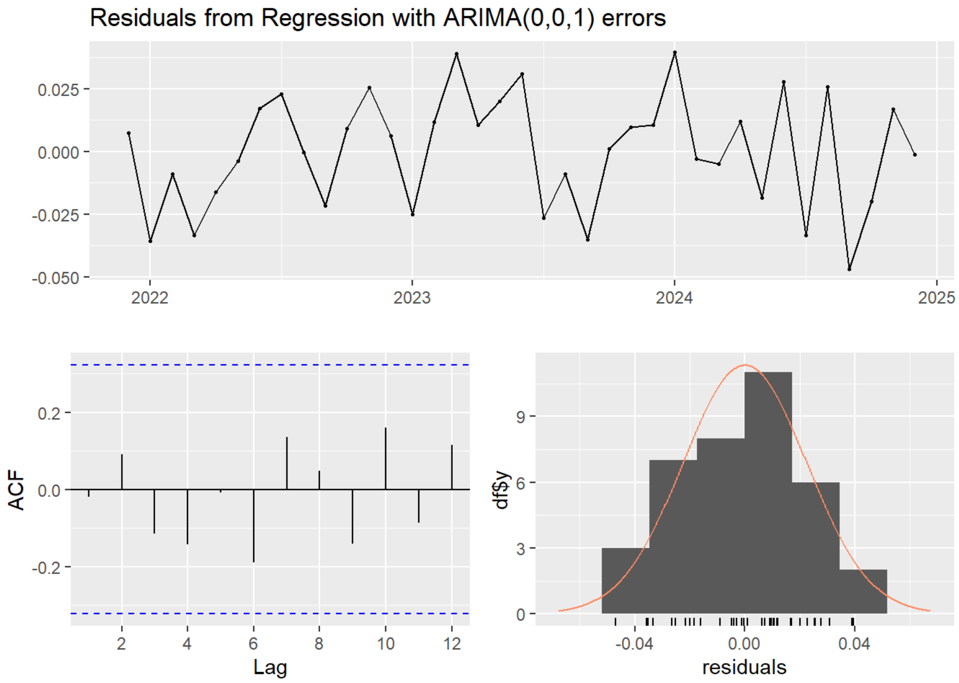

Figure S2: Residuals time series plot, residuals auto-correlation function (ACF) plot, and residuals histogram for unadjusted ARIMA(0,0,1) model for the outcome of prevalence of vape use in past-year quit attempts.

- Ljung-Box test results for residuals from ARIMA(0,0,1) for the outcome of prevalence of vape use in past-year quit attempts with non-zero mean: Q*=4.12, df=6, p=0.661 → no significant autocorrelation.
- Adjusted model: ARIMA(0,0,1) with dummy variable for tobacco tax increases using a step change to model the intervention effect of the implementation of the Swap to Stop programme

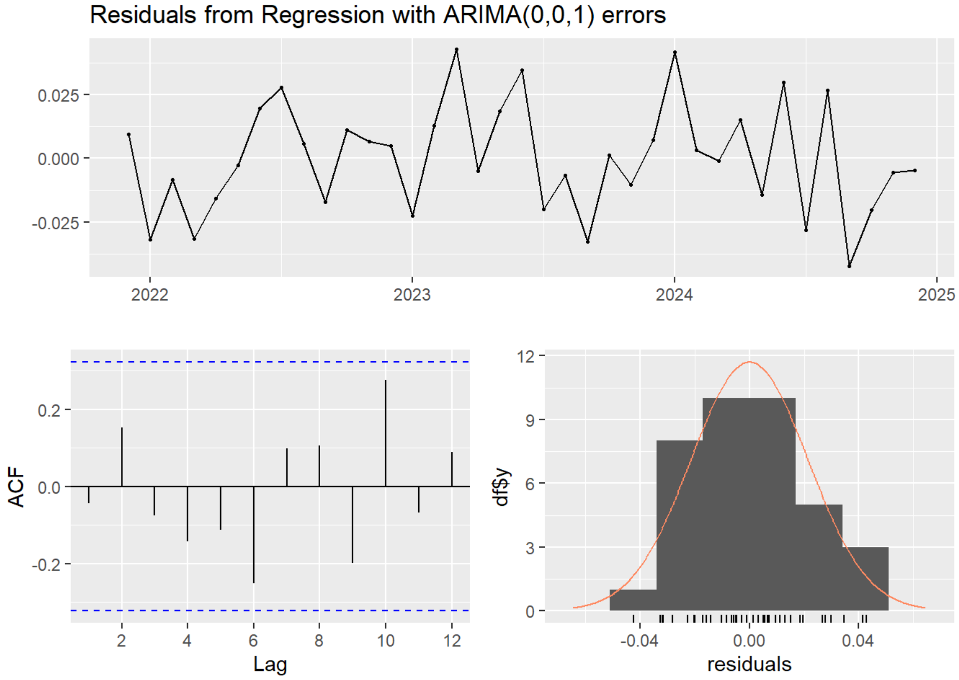

Figure S3: Residuals time series plot, residuals auto-correlation function (ACF) plot, and residuals histogram for adjusted ARIMA(0,0,1) model for the outcome of prevalence of vape use in past-year quit attempt as the outcome variable.

- Ljung-Box test results for residuals from adjusted ARIMA(0,0,1) for the outcome of prevalence of vape use in past-year quit attempt and adjustment for tax increases with non-zero mean: Q*=6.13, df=6, p=0.409 → no significant autocorrelation.

Table S5: Best fitting model for prevalence of vape use in past-year quit attempt as the outcome variable – ARIMA(0,0,1).

Variables	Unadjusted model		Adjusted model	
	B (95% CI)	p-value	B (95% CI)	p-value
MA1	-0.238 (-0.546, 0.070)	0.131	-0.324 (-0.633, -0.014)	0.040
Intercept	0.132 (0.125, 0.138)	<0.001	0.129 (0.123, 0.135)	<0.001
Swap to Stop (intervention effect modelled using a step change)	0.014 (0.003, 0.026)	0.017	0.015 (0.005, 0.025)	0.004
Tax increases (comprised score as dummy variable)	–	–	0.023 (0.000, 0.046)	0.053

Sensitivity analysis 1a: alternative step change

Pre-planned sensitivity analysis with alternative step change in April 2024.

Table S6: Results for sensitivity analysis using alternative step change with model ARIMA(0,0,1) and covariates the same as in the main analysis, with prevalence past-year vape use in quit attempts as the outcome variable.

Variables	Unadjusted model		Adjusted model	
	B (95% CI)	p-value	B (95% CI)	p-value
MA1	-0.147 (-0.440, 0.145)	0.322	-0.203 (-0.494, 0.087)	0.170
Intercept	0.135 (0.127, 0.142)	<0.001	0.133 (0.125, 0.140)	<0.001
Swap to Stop (intervention effect modelled using a step change)	0.008 (-0.007, 0.023)	0.299	0.008 (-0.006, 0.022)	0.263
Tax increases (comprised score as dummy variable)	–	–	0.018 (-0.006, 0.043)	0.142

The Box-Ljung test indicates no significant autocorrelation for the time-series in the unadjusted (Q*=4.80, df=6, p=0.570) and adjusted model (Q*=5.94, df=6, p=0.430).

Sensitivity analysis 1b: alternative outcome

Pre-planned sensitivity analysis with alternative outcome – prevalence of past-month vape use in quit attempts.

Table S7: Results for sensitivity analysis with model ARIMA(0,0,1) and covariates the same as in the main analysis, with prevalence past-month vape use in quit attempts as the outcome variable.

Variables	Unadjusted model		Adjusted model	
	B (95% CI)	p-value	B (95% CI)	p-value
MA1	-0.102 (-0.403, 0.199)	0.507	-0.167 (-0.476, 0.142)	0.289
Intercept	0.095 (0.088, 0.103)	<0.001	0.094 (0.087, 0.101)	<0.001
Swap to Stop (intervention effect modelled using a step change)	0.009 (-0.003, 0.021)	0.151	0.009 (-0.002, 0.020)	0.096
Tax increases (comprised score as dummy variable)	–	–	0.016 (-0.006, 0.037)	0.148

The Box-Ljung test indicates no significant autocorrelation for the time-series in the unadjusted (Q*=7.50, df=6, p=0.277) and adjusted model (Q*=5.89, df=6, p=0.435).

Association between age and using vapes in quit attempts

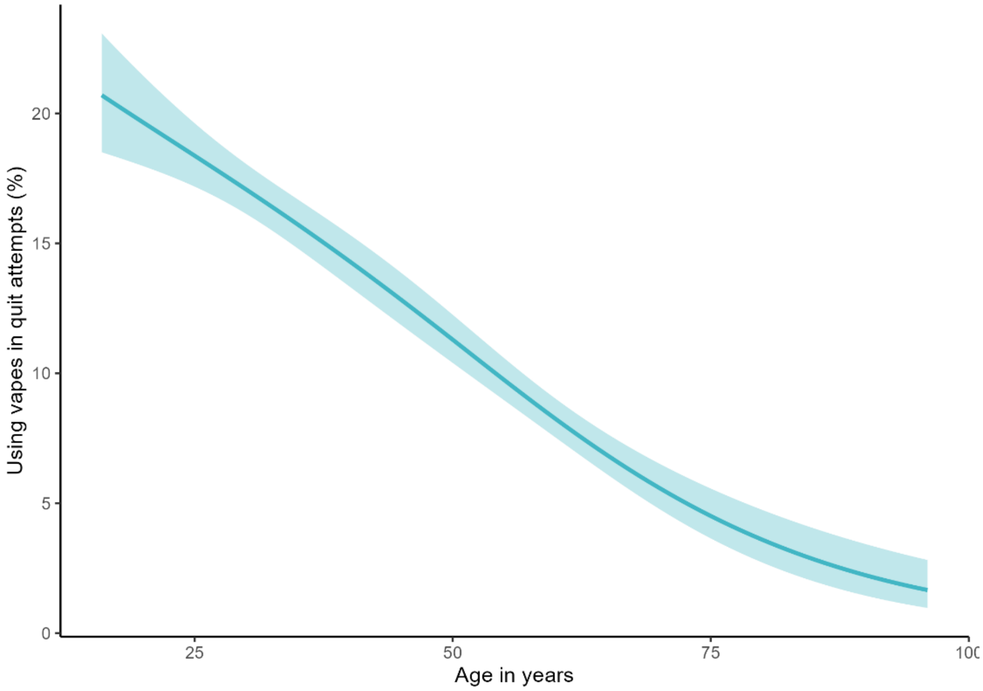

Figure S4: Weighted association between age and using vapes in quit attempts, based on all who smoked in the past year in Great Britain across the entire study period. Age modelled using restricted cubic splines with 3 knots (minimum, median, and maximum age).

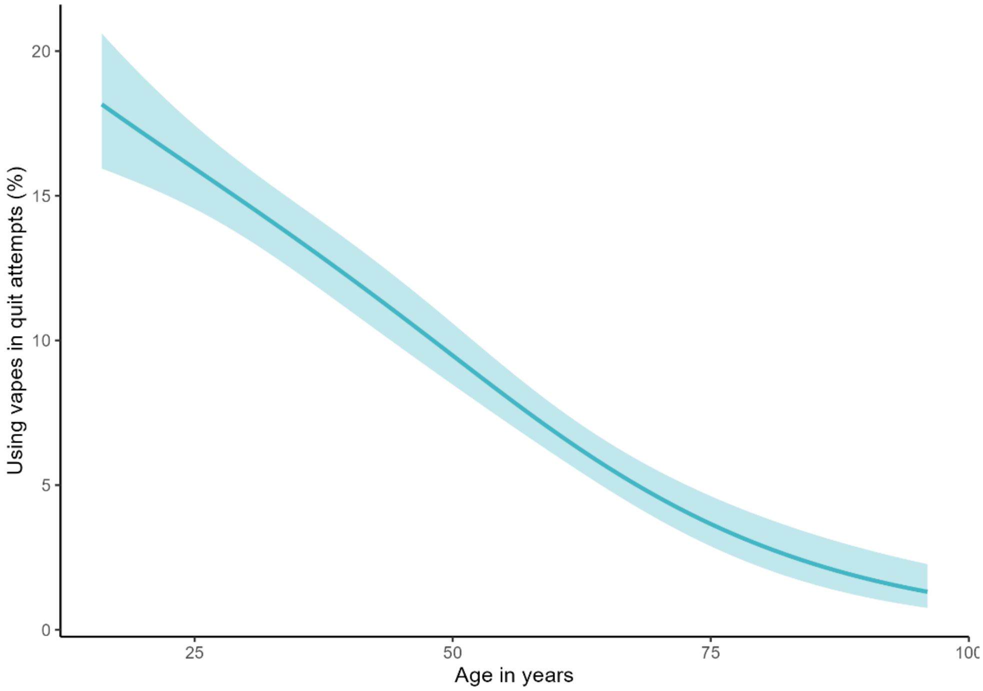

Figure S5: Weighted association between age and using vapes in quit attempts adjusted for gender and social grade, based on all who smoked in the past year in Great Britain across the entire study period. Age modelled using restricted cubic splines with 3 knots (minimum, median, and maximum age).

Difference-in-differences – vape use in past-year quit attempts

Model specifications:

- Generalised Additive Model (GAM) using gam-function of mgcv package [15]
- Family: quasibinomial; link function: log; smooth term: cyclic cubic regression spline
- Method: restricted maximum likelihood; optimizer: outer newton
- Formula (only adjusted for seasonality): past-year vape use in quit attempt ~ intervention*step + intervention*time + s(seasonality, k = 12)

Table S8: Parameter estimates for GAM with prevalence of vape use in past-year quit attempt as outcome.

**Variables**	**Unadjusted[1] model**			**Adjusted[2] model**		
	B (95% CI)	p-value	OR (95% CI)	B (95% CI)	p-value	OR (95% CI)
Baseline average	-1.982 (-2.293, -1.671)	<0.001	0.14 (0.10, 0.19)	-1.675 (-2.004, -1.345)	<0.001	0.19 (0.13, 0.26)
Time trend in Scotland/Wales	-0.014 (-0.037, 0.009)	0.246	0.99 (0.96, 1.01)	-0.009 (-0.032, 0.014)	0.459	0.99 (0.97, 1.01)
Difference between groups before December 2023	-0.055 (-0.387, 0.276)	0.743	0.95 (0.68, 1.32)	0.110 (-0.437, 0.217)	0.509	0.9 (0.65, 1.24)
Difference in England before and after December 2023	0.202 (-0.341, 0.745)	0.466	1.22 (0.71, 2.11)	0.194 (-0.345, 0.733)	0.418	1.21 (0.71, 2.08)
Difference in time trend in England before and after December 2023	0.016 (-0.009, 0.040)	0.213	1.02 (0.99, 1.04)	0.010 (-0.014, 0.034)	0.414	1.01 (0.99, 1.03)
Difference-in-differences: between groups before and after December 2023	-0.127 (-0.696, 0.441)	0.661	0.88 (0.50, 1.55)	-0.105 (-0.664, 0.454)	0.713	0.90 (0.51, 1.57)

[1]Adjusted for seasonality. [2]Adjusted for seasonality, tobacco tax increases, age, gender, social grade. Abbreviation: OR, odds ratio.

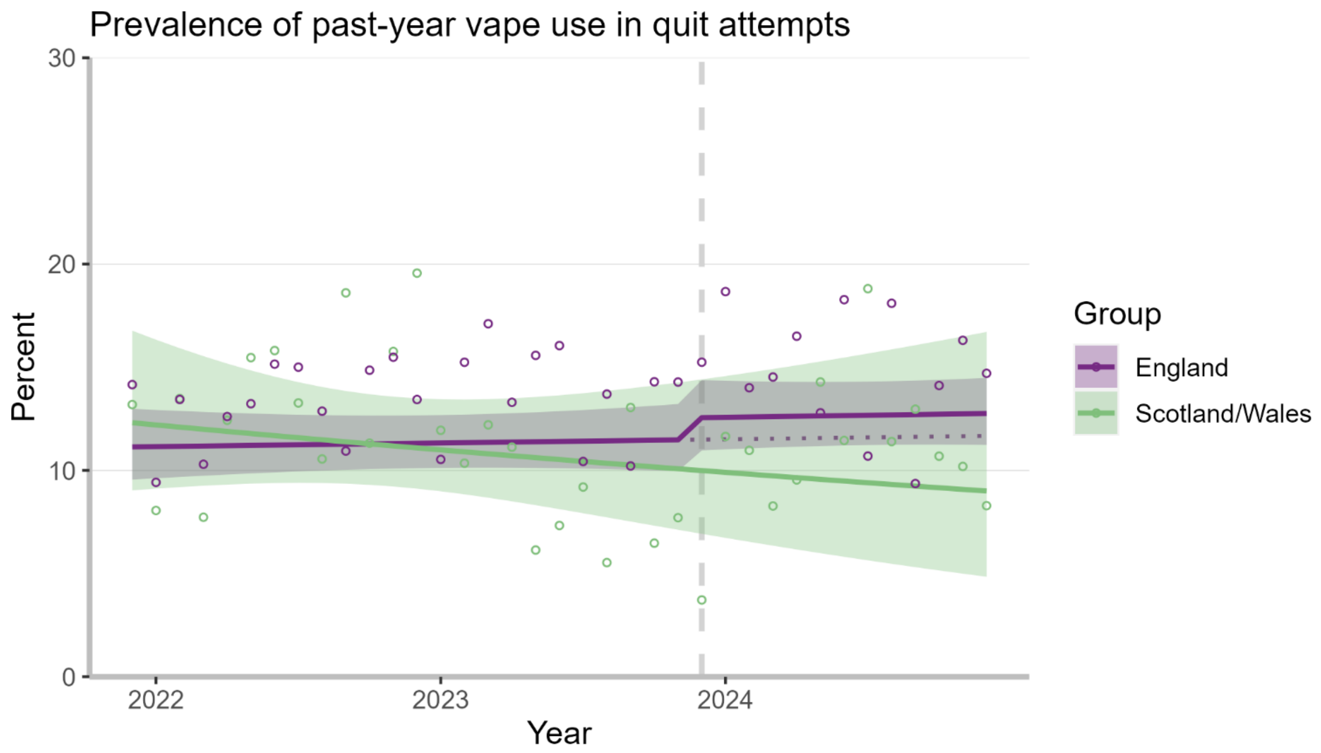

Figure S6: Modelled trends before and after December 2023 (start of Swap to Stop) in England (intervention group) compared to Scotland and Wales (control group), adjusted for seasonality, tobacco tax increases, age, and social grade. Dashed vertical line shows start of Swap to Stop programme (intervention). Dotted line indicates continued pre-intervention trend in England. Shaded areas indicate 95% CIs and dots unmodelled estimates.

References

1 Hyndman RJ, Khandakar Y. Automatic time series forecasting: the forecast package for R. *Journal of Statistical Software* 2008;27(3):1 - 22. doi: 10.18637/jss.v027.i03

2 Beard E, Marsden J, Brown J, et al. Understanding and using time series analyses in addiction research. *Addiction* 2019;114(10):1866-84. doi: 10.1111/add.14643

3 Metes DV. Visual, unit root and stationarity tests and their power and accuracy. Edmonton, Canada: Department of Mathematical and Statistical Sciences, University of Alberta, 2005.

4 Dickey DA, Fuller WA. Distribution of the estimators for autoregressive time series with a unit root. *Journal of the American statistical association* 1979;74(366a):427-31. doi: 10.1080/01621459.1979.10482531

5 Wang X, Smith K, Hyndman R. Characteristic-based clustering for time series data. *Data Mining and Knowledge Discovery* 2006;13(3):335-64. doi: 10.1007/s10618-005-0039-x

6 Wickham H, Averick M, Bryan J, et al. Welcome to the tidyverse. *Journal of Open Source Software* 2019;4(43):1686. doi: 10.21105/joss.01686

7 Survey: analysis of complex survey samples [program]. R package version 4.0 version. CRAN R-project, 2020.

8 tibbletime: time aware tibbles [program]. R package version 0.1.6 version. CRAN R-Project, 2020.

9 Hyndman RJ, Athanasopoulos G, Bergmeir C, et al. forecast: Forecasting functions for time series and linear models, 2023.

10 Wickham H. ggplot2: elegant graphics for data analysis. New York, United States: Springer-Verlag 2016.

11 tseries: time series analysis and computational finance [program]. R package version 0.10-54 version. CRAN R-project, 2023.

12 Chan K-S, Ripley B. Time Series Analysis - Package 'TSA'. CRAN repository, 2022.

13 zoo: S3 Infrastructure for Regular and Irregular Time Series (Z's Ordered Observations) [program]. R package version 1.8-13 version. CRAN R-Project, 2025.

14 R-Core Team. splines (version 3.6.2): RDocumentation; 2019; updated 12 December 2019. https://rdocumentation.org/packages/splines/versions/3.6.2 (accessed 25 March 2025).

15 Wood SN. mgcv: Mixed GAM Computation Vehicle with Automatic Smoothness Estimation Vienna, Austria: CRAN Repository; 2023; updated 21 December 2021. https://cran.r-project.org/web/packages/mgcv/index.html (accessed 20 June 2024).
